# Supplementary figures and images for: Gut microbiota features associated with Clostridioides difficile colonization in dairy calves
Source: PLoS One. 2021 Dec 15;16(12):e0251999. doi: 10.1371/journal.pone.0251999 (PMC8673638; doi:10.1371/journal.pone.0251999)

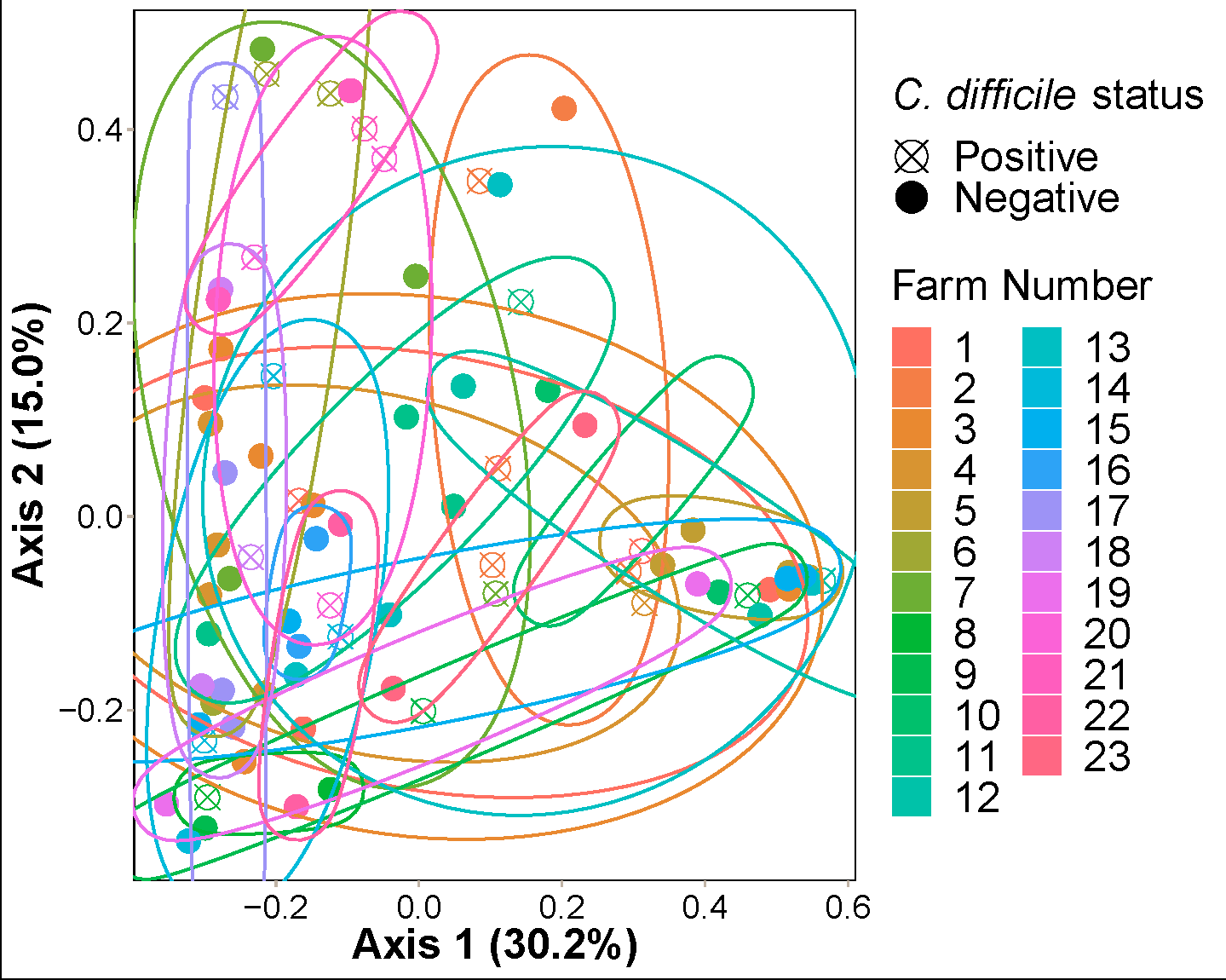

Supplement: S1 Fig — Point shape identifies C. difficile colonization status and color denotes farm. 95% confidence intervals were drawn around samples collected in the same farm. (TIF) [file pone.0251999.s001.tif]
